# Under pressure: design and validation of a pressure-sensitive insole for ankle plantar flexion biofeedback during neuromuscular gait training

**DOI:** 10.1186/s12984-022-01119-y

**Published:** 2022-12-08

**Authors:** Benjamin C. Conner, Ying Fang, Zachary F. Lerner

**Affiliations:** 1 College of Medicine–Phoenix, University of Arizona, Phoenix, AZ USA; 2 Department of Mechanical Engineering, Northern Arizona University, 15600 S McConnell Drive, NAU EGR Bldg 69, Flagstaff, AZ 86011 USA

**Keywords:** Cerebral palsy, Force sensitive resistor, Plantar pressure, Neurorehabilitation, Audiovisual

## Abstract

**Background:**

Electromyography (EMG)-based audiovisual biofeedback systems, developed and tested in research settings to train neuromuscular control in patient populations such as cerebral palsy (CP), have inherent implementation obstacles that may limit their translation to clinical practice. The purpose of this study was to design and validate an alternative, plantar pressure-based biofeedback system for improving ankle plantar flexor recruitment during walking in individuals with CP.

**Methods:**

Eight individuals with CP (11–18 years old) were recruited to test both an EMG-based and a plantar pressure-based biofeedback system while walking. Ankle plantar flexor muscle recruitment, co-contraction at the ankle, and lower limb kinematics were compared between the two systems and relative to baseline walking.

**Results:**

Relative to baseline walking, both biofeedback systems yielded significant increases in mean soleus (43–58%, p < 0.05), and mean (68–70%, p < 0.05) and peak (71–82%, p < 0.05) medial gastrocnemius activation, with no differences between the two systems and strong relationships for all primary outcome variables (*R* = 0.89–0.94). Ankle co-contraction significantly increased relative to baseline only with the EMG-based system (52%, p = 0.03).

**Conclusion:**

These findings support future research on functional training with this simple, low-cost biofeedback modality.

**Supplementary Information:**

The online version contains supplementary material available at 10.1186/s12984-022-01119-y.

## Background

Effective recruitment of the ankle plantar flexor muscles is necessary to modulate the forward and vertical progression of the center of mass for an efficient exchange of potential and kinetic energy during bipedal walking [1, 2]. Individuals with cerebral palsy (CP) [3], stroke [4, 5], and the elderly [6], often lack the neuromuscular control to effectively utilize their plantar flexors during walking. For individuals with CP, the most prevalent pediatric-onset movement disorder, there is broad clinical agreement that plantar flexor dysfunction often contributes to gait impairment [7], creating a barrier to an active lifestyle and predisposing this population to a host of secondary effects associated with inactivity [8], including an eventual loss of independent ambulation [9]. For this reason, interventions designed to improve neuromuscular control of the ankle plantar flexors could have a significant impact on long-term mobility for individuals with CP, or any other patient populations that experience reduced motor control at the ankle.

Several audiovisual biofeedback systems (e.g., step-length feedback) have been developed for individuals with CP with the goal of modulating upper or lower limb position, force, or motor control [10, 11]. To date, most audiovisual biofeedback studies aimed at increasing lower-limb muscle control in CP have utilized an electromyography (EMG)-based system, whereby a user’s muscle activity is displayed to them in real-time [12–14]. While EMG-based audiovisual biofeedback provides direct feedback of the intervention’s target (i.e., increased muscle activity), there are significant limitations to the EMG biofeedback modality that prevents widespread adoption in clinical or home settings, including motion artifact noise during walking; skin-electrode interface reliability challenges, like hair and sweating; the necessity and complexity of proper anatomical placement of the sensors, particularly when placing sensors on small limbs; and the cost of an EMG system. This may explain why, despite a demonstrated benefit of plantar flexor EMG-based biofeedback for improving ankle function and gait symmetry in CP nearly three decades ago, this gait training tool has failed widespread adoption in clinical practice. Practical biofeedback modalities capable of increasing plantar flexor recruitment during gait training would likely have widespread appeal.

We theorize that a potential alternative to a plantar flexor EMG-based biofeedback system could be an underfoot plantar pressure-based system that would measure and provide feedback on the change in forefoot pressure generated from plantar flexor muscle recruitment. Pressure sensors are inexpensive and could be quickly and easily accommodated by most footwear, and have been used previously to modulate muscle activity at the ankle during walking for individuals with chronic ankle instability [15]. If effective, plantar pressure-based biofeedback may expand access to neuromuscular gait training by offering a practical solution for in-clinic and at-home use. Before a plantar pressure-based system like this could be clinically translated, however, it should be validated by comparing changes in muscle activity with those observed from an EMG-based system during walking.

The primary aim of this study was to clinically validate the use of a plantar pressure-based audiovisual biofeedback system to increase ankle plantar flexor engagement during walking by comparing changes in muscle activation levels to an EMG-based audiovisual biofeedback system in CP. We hypothesized that both biofeedback modalities would result in a significant increase in plantar flexor activity while walking, with no difference and strong relationships between the two systems, validating the use of the plantar pressure-based system as an alternative to an EMG-based system.

## Methods

**Fig. 1:**
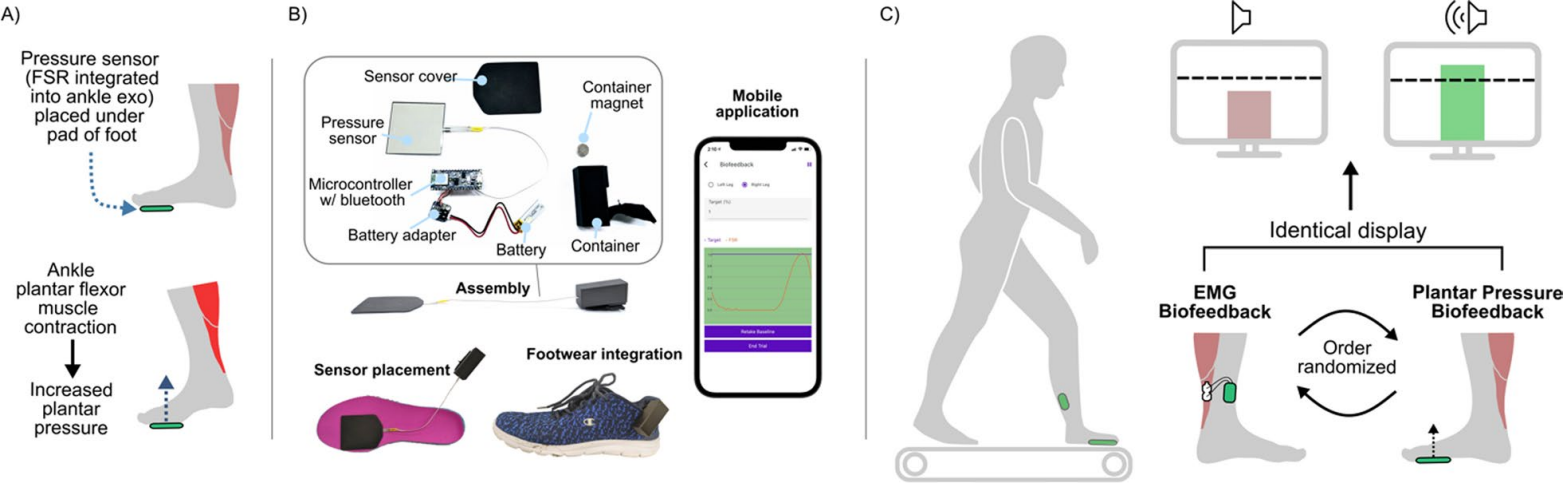
**A** Plantar pressure-based setup, with a force sensitive resistor (FSR) placed under the pad of the foot to measure changes in plantar pressure with contraction of the ankle plantar flexors; **B** standalone plantar pressure-based biofeedback system; **C** experimental protocol for validating the plantar pressure-based audiovisual biofeedback system against an EMG-based system (measuring and displaying soleus muscle activity), where both systems used an identical display of a real-time moving bar graph and horizontal target line with an audible “ding” when this target line was passed

We developed a simple plantar pressure-based biofeedback system comprised of a force sensitive resistor (FSR) placed under the ball of the fore-foot that responded to plantar flexor muscle activity indirectly through push-off with the ground (Fig. 1A). We compared our plantar pressure-based system to EMG-based biofeedback, which we considered the “gold standard”, given that it provides direct feedback of the metric that a user is attempting to modulate (i.e., muscle activation), and has been tested in previous studies [13, 16]. To isolate the effects of each biofeedback modality while controlling for the components attached to the body, we used the same wearable setup (i.e., plantar pressure and EMG systems) during all walking conditions (i.e., baseline, plantar pressure-based biofeedback, and EMG-based biofeedback).

### Plantar pressure biofeedback system

Plantar pressure was measured using an FSR (FlexiForce A502, Tekscan) secured to a thin carbon fiber foot plate. The FSR had a relatively large (51 × 51 mm) sensing area that was centered under the typical location for the head of the first metatarsal (the “pad” or “ball” of the foot). The FSR location remained the same for each participant, as an aspect of the present study was to evaluate if an FSR placed in this area could serve as a suitable metric for providing audiovisual biofeedback of ankle plantar flexion for different foot types. The FSR was wired to a custom printed circuit board with a microcontroller and Bluetooth transceiver, utilizing electronics from a modified version of our wearable robotic system presented in [17]. In this study, the Bluetooth transceiver communicated with a laptop running a real-time MATLAB graphical user interface (GUI). To demonstrate the broader applicability of our approach, we also created a stand-alone system with a smartphone application (available for iOS and Android from Biomotum, Inc.). The stand-alone system, weighing < 50 g, had the components condensed into a portable casing that can be attached to a user’s footwear (Fig. 1B).

A 5-s calibration procedure was completed at the beginning of each walking trial. The instantaneous plantar pressure data were then normalized by the average peak stance phase pressure during the calibration period and transmitted at 100 Hz to a moving bar graph on the MATLAB GUI. A “target” horizontal line was also included on the bar graph, which was initially set to 10% above the average peak plantar pressure from the calibration period. The target was then adjusted based on each user’s performance. Specifically, if a user reached their target greater than 75% of steps within a minute, the line was increased by 10%. If a user reached their target less than 50% of steps within a minute, the line was decreased by 10%. If a user reached their target between 50 and 75% of steps within a minute, the line was held constant. If a participant reached the target line, the bar graph changed from red to green, and a “ding” sound was emitted from the laptop speakers. Once the bar dropped back below the target line, the bars changed back to red (Fig. 1C). Biofeedback was provided for each participant’s more impaired limb.

### EMG biofeedback system

We used a commercially available system for EMG-based biofeedback (Desktop DTS, Noraxon), which displayed real-time soleus muscle activity after filtering with a 1000 ms root mean square envelope. The filtered muscle activity was presented in an identical display as the plantar pressure-based system; the same auditory feedback was also provided. To match the plantar pressure-based system, a “target” horizontal line was also set at 10% above a user’s baseline peak soleus activity, with the same performance-based adjustments to this target as the plantar pressure system (Fig. 1C). Feedback was provided on each participant’s more impaired limb.

### Participants

This protocol was approved by the Northern Arizona University Institutional Review Board (#986744) and completed at the Northern Arizona University–Phoenix Biomedical Campus (Phoenix, AZ). The protocol utilized participants recruited for a clinical trial, which was prospectively registered at ClinicalTrials.gov (NCT04119063). Prior to starting the study, informed written consent was provided by each participant if 18 years or older, or the parent/legal guardian in the case of minors (minors also provided verbal assent).

Participant inclusion criteria was as follows: confirmed diagnosis of CP (hemi- or diplegic distributions), Gross Motor Function Classification System (GMFCS) level I–III, the ability to walk on a treadmill with support for at least ten minutes, and 10–21 years of age. Exclusion criteria included orthopedic surgery within the past 6 months, botulinum toxin injections to the triceps surae muscles within the past 6 months, and any other conditions that would preclude safe participation. Eight participants were recruited (Table 1).

**Table 1:** Participant characteristics

	Gender	Age (y)	Height (m)	Weight (kg)	GMFCS	Distribution
P1	M	13	1.48	38.6	II	Diplegic
P2	M	15	1.58	59.4	II	Diplegic
P3	F	11	1.43	41.7	III	Diplegic
P4	M	13	1.58	38.5	III	Diplegic
P5	M	16	1.61	48.5	II	Diplegic
P6	M	18	1.75	60.3	II	Diplegic
P7	M	17	1.65	65.8	I	Hemiplegic
P8	M	12	1.40	39.5	I	Hemiplegic

*GMFCS* Gross Motor Function Classification System level

### Protocol

To begin, height, weight, and lower limb anthropometrics were measured for each participant. Next, participants were outfitted with reflective markers on their lower limbs in accordance with Vicon’s lower body Plug-In Gait model (Vicon, 100 Hz; Denver, CO, USA), and wireless surface EMG sensors (Noraxon, 1000 Hz; Scottsdale, AZ, USA) were placed on the more affected limb’s soleus, medial gastrocnemius, and tibialis anterior muscles according to SENIAM recommendations [18]. For participants who were diplegic, the more affected limb was determined by asking the participant or participant’s guardian which side was weaker and/or less coordinated. All participants and/or participant guardians were able to clearly identify a more affected side.

Participants walked under the following three conditions at a self-selected speed on the treadmill for two minutes and 30 s while lower body kinematics and muscle activity were measured: (1) baseline (i.e., no audiovisual biofeedback), (2) unilateral plantar pressure-based audiovisual biofeedback, and (3) unilateral EMG-based audiovisual biofeedback. All participants started with the baseline condition, followed by the plantar pressure and EMG biofeedback conditions in random order. P1, a younger participant, was re-tested on a separate day because of an apparent behavior non-compliance issue.

### Data analysis

Twenty gait cycles of each condition were analyzed. Three-dimensional marker data was used to calculate ankle and knee joint angles for both biofeedback conditions versus baseline using Vicon’s Plug-In Gait Model and inverse kinematics. Angle data was low-pass filtered (4th order Butterworth, 4 Hz low-pass cutoff) and an average curve for both ankle angle and knee angle was generated. EMG data was bandpass filtered (4th order Butterworth, 20–400 Hz band-pass cutoff), rectified, and low-pass filtered (4th order Butterworth, 4 Hz low-pass cutoff [19]), and then time normalized to a gait cycle (heel strike to heel strike). The twenty recorded gait cycles were averaged together for a single activation curve for each muscle, and normalized to peak baseline activation of that respective muscle.

Peak stance phase ankle plantar flexion and knee extension angles were calculated for each condition. We chose to assess lower limb kinematics to determine if either system resulted in compensatory strategies that would negatively impact gait. In addition, mean and peak propulsive phase (51–100% of stance phase [20]) soleus and medial gastrocnemius activation was calculated for each condition. Finally, stance-phase co-contraction at the ankle between the soleus and tibialis anterior was calculated for each condition using a co-contraction index (CCI) [21]:

1 $$\text{CCI} = \sum_{i=1}^{101}\frac{LEMG\,(i)}{MEMG\,(i)} \left(LEMG\,(i)+MEMG\,(i)\right)$$

where *i* represents the individual time points of stance phase (0–100%, or 101 total data points), *LEMG* represents the normalized magnitude of the less active muscle at time point *i*, and *MEMG* represents the normalized magnitude of the more active muscle at time point *i*. CCI values for the audiovisual biofeedback conditions were then normalized to baseline values.

### Statistical analysis

We validated plantar pressure-based biofeedback through three main statistical comparisons: (1) by assessing the change in muscle activity relative to baseline; (2) by comparing the change in muscle activity relative EMG biofeedback; and (3) similar to other validation studies [22, 23], by assessing the relationship to EMG-based biofeedback.

The primary outcome measures for our *a-priori* hypotheses included mean and peak propulsive phase soleus and medial gastrocnemius muscle activity. Secondary outcome measures included ankle CCI, and peak ankle plantar flexion and knee extension angle. To assess our primary objective of validating plantar pressure vs. EMG biofeedback, we performed one-way repeated measures Analysis of Variance (RM ANOVA) to determine the effect of biofeedback condition (i.e., baseline, plantar pressure biofeedback, and EMG biofeedback) on these outcome measures. If a significant effect of walking condition was found, we ran two-tailed pairwise comparisons with Holm–Bonferroni correction for multiple comparisons. In addition, we assessed the relationship between the plantar pressure-based and EMG-based primary outcome measures by calculating a Pearson product-moment correlation coefficient (*R*), where 0.3 was considered a weak relationship, 0.5 a moderate relationship, and 0.7 a strong relationship [24]. To ensure that outlier values did not significantly influence this relationship, we ran an outlier analysis where any values 1.5 times the interquartile range below the first quartile or above the third quartile were removed the correlation calculation [25]. P7’s medial gastrocnemius data was not available for the primary objective due to a dropped signal from the EMG sensors. All reported p-values are the adjusted values from the multiple comparisons correction, and significance level was set at p < 0.05.

## Results

All participants successfully completed the protocol, walking with both audiovisual biofeedback systems and understanding their objective of reaching their target line on each step.

**Fig. 2:**
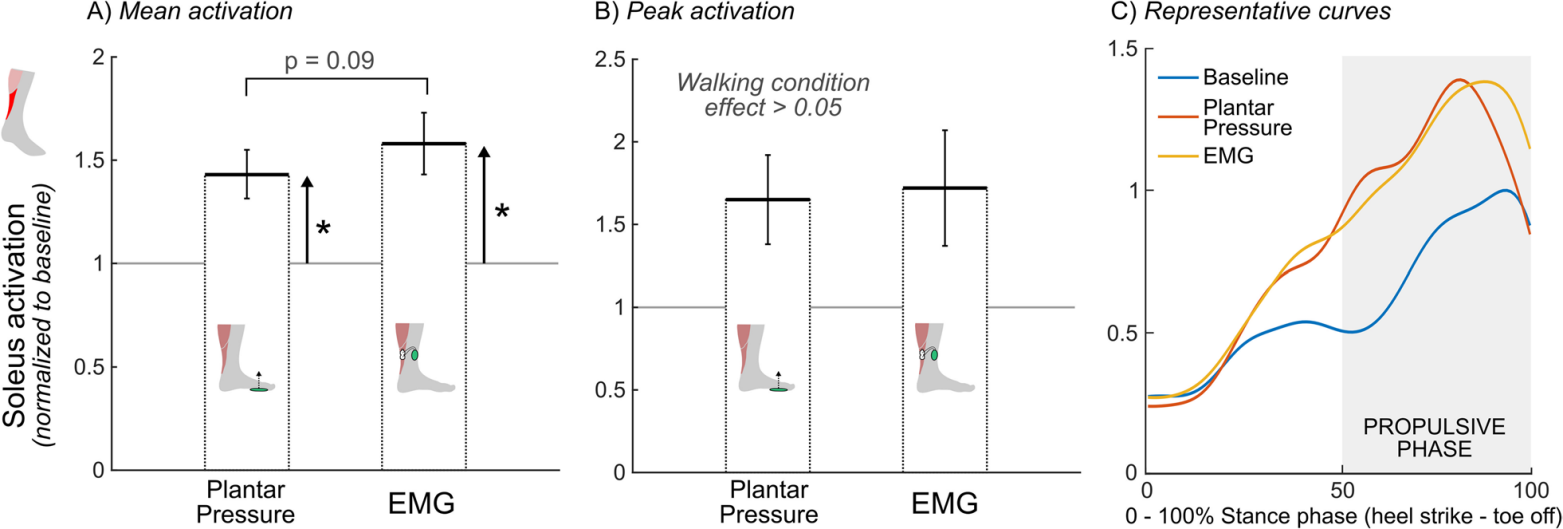
Average **A** mean and **B** peak propulsive phase soleus activation relative to baseline activity for the two audiovisual biofeedback systems, and **C** representative soleus activation curves (average of 20 gait cycles) across the three walking conditions: baseline (blue), plantar pressure-based biofeedback (orange), and EMG-based biofeedback (yellow); Error bars represent standard error of the mean, brackets indicate pairwise comparisons between plantar pressure-based and EMG-based systems, upward arrows represent pairwise comparisons between baseline walking and the respective audiovisual biofeedback system; *p < 0.05

**Fig. 3:**
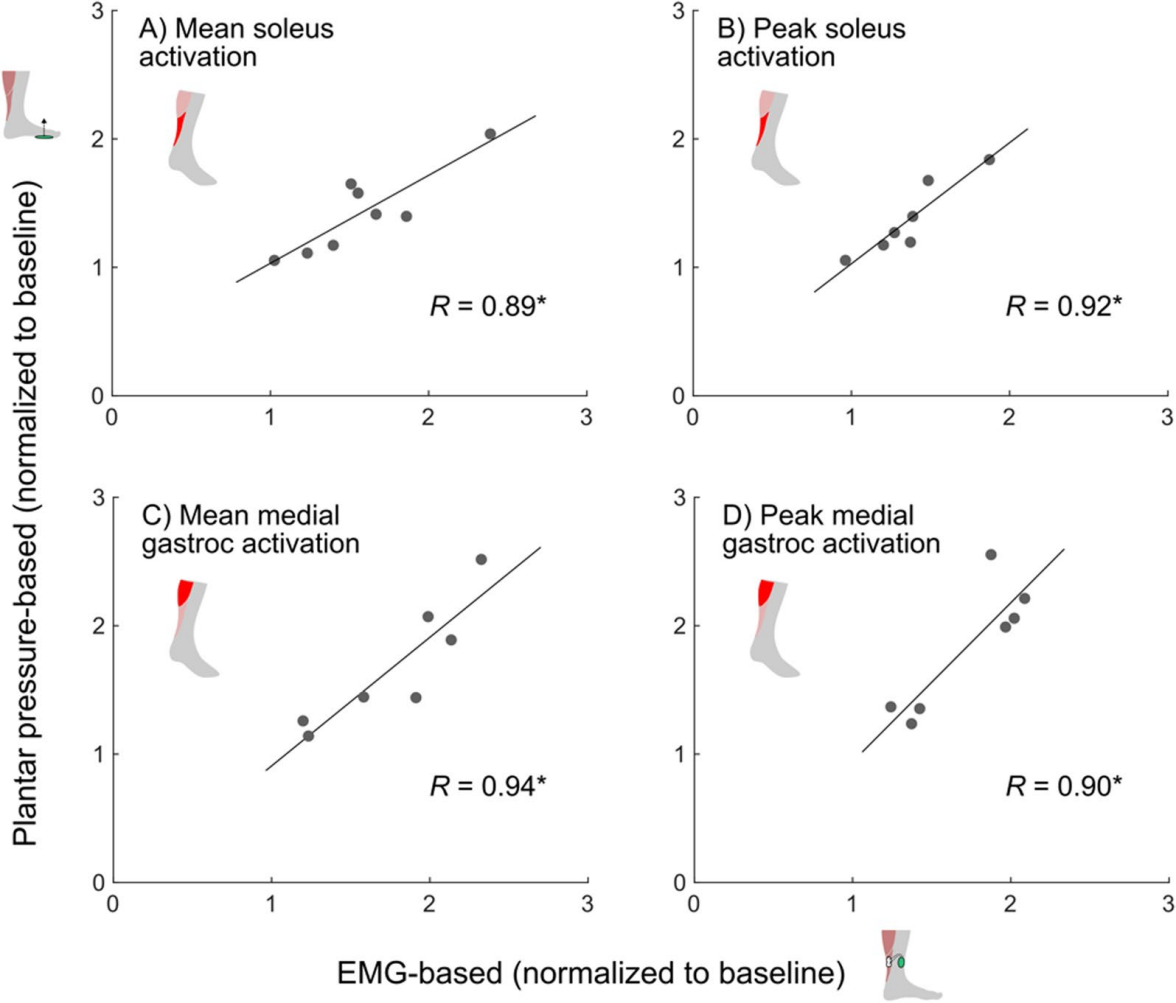
Relationships between the EMG-based and plantar pressure-based systems for **A** mean soleus activation, **B** peak soleus activation, **C** mean medial gastrocnemius activation, and **D** peak medial gastrocnemius activation, where all values were normalized to baseline; *p < 0.05

There was a significant effect of walking condition on mean soleus activity (p < 0.01; Fig. 2A). Pairwise comparisons indicated a significant increase in mean soleus activity for both the plantar pressure-based (mean ± SD) (43 ± 33%, p < 0.05) and EMG-based systems (58 ± 42%, p < 0.05) relative to mean baseline soleus activity, with no difference between the two systems (p = 0.09). There was no significant effect of walking condition on peak soleus activity (p = 0.08; Fig. 2B). Strong relationships were observed between the two systems for both mean (*R* = 0.89, p < 0.01, Fig. 3A) and peak (*R* = 0.92, p < 0.01, Fig. 3B) soleus activation. See Additional file 1 for individual soleus activation curves for these conditions.

**Fig. 4:**
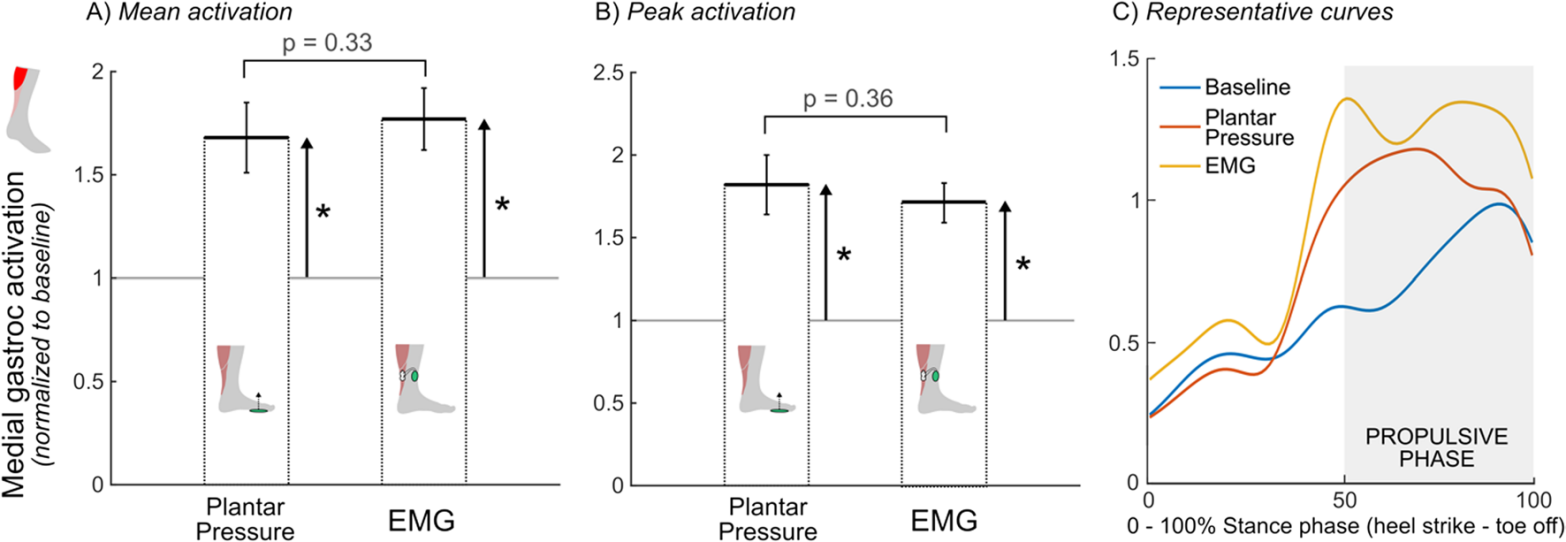
Average **A** mean and **B** peak propulsive phase medial gastrocnemius activation relative to baseline activity for the two audiovisual biofeedback systems, and **C** representative medial gastrocnemius activation curves (average of 20 gait cycles) across the three walking conditions: baseline (blue), plantar pressure-based biofeedback (orange), and EMG-based biofeedback (yellow); Error bars represent standard error of the mean, brackets indicate pairwise comparisons between plantar pressure-based and EMG-based systems, upward arrows represent pairwise comparisons between baseline walking and the respective audiovisual biofeedback system; *p < 0.05

A significant effect of walking condition was observed for both mean (p < 0.01; Fig. 4A) and peak (p < 0.01; Fig. 4B) medial gastrocnemius activity. Pairwise comparisons indicated a significant increase in mean medial gastrocnemius activation for both the plantar pressure-based (68 ± 50%; p < 0.05) and EMG-based (77 ± 44%; p < 0.05) systems relative to baseline, with no difference between systems (p = 0.33). Additionally, both systems had a significant increase in peak medial gastrocnemius activation relative to baseline (plantar pressure-based: 82 ± 51%, p < 0.05; EMG-based: 71 ± 35%, p < 0.01), but no difference was observed between systems (p = 0.36). There were strong relationships between the two systems for both mean (*R* = 0.94, p < 0.01, Fig. 3C) and peak (*R* = 0.90, p < 0.01, Fig. 3D) medial gastrocnemius activation. See Additional file 2 for individual medial gastrocnemius activation curves for these conditions.

**Fig. 5:**
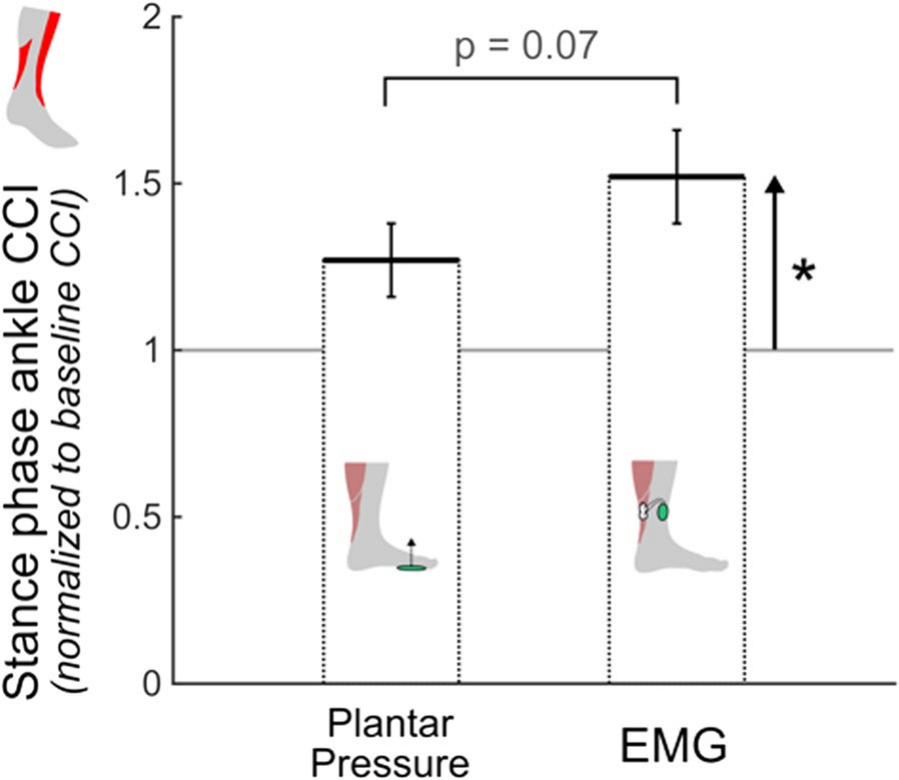
Average co-contraction index (CCI) between the soleus and tibialis anterior during the stance phase of gait for the two audiovisual biofeedback systems alone; Error bars represent standard error of the mean, brackets indicate pairwise comparisons between plantar pressure-based and EMG-based systems, and upward arrows represent pairwise comparisons between baseline walking and the respective audiovisual biofeedback system

A significant effect of walking condition on ankle CCI was found (p < 0.01; Fig. 5); pairwise comparisons indicated a significant increase for the EMG-based system relative to baseline walking (52 ± 41%, p = 0.03), but no difference between the plantar pressure-based system and baseline walking (p = 0.07) or between the two systems (p = 0.07). No significant effects of walking condition were observed for the lower limb kinematic measures (peak knee extension angle: p = 0.6, peak ankle plantar flexion angle: p = 0.5; see Additional file 3 for individual joint angle curves).

## Discussion

We achieved our primary goal of validating a plantar pressure biofeedback system for increasing ankle plantar flexor muscle activity relative to EMG biofeedback, and demonstrated several expected and one surprising potential benefit of our novel system compared to the “gold-standard.” The findings from this study partially supported our hypothesis that both plantar pressure-based and EMG-based audiovisual biofeedback systems would increase ankle plantar flexor muscle activity while walking, with significant increases in mean soleus muscle activity and mean and peak medial gastrocnemius muscle activity relative to baseline for both systems, and trends toward increases in peak soleus activity. Importantly, there was no statistical difference in activation between the two biofeedback modalities, and strong relationships for all primary outcome variables between the two systems. Our finding of consistent lower limb gait kinematics across both biofeedback conditions, and relative to baseline walking, indicated that participants did not adopt any compensatory movement strategies in response to feedback that could negatively affect their gait.

While an ankle plantar flexor EMG-based audiovisual biofeedback system provides direct feedback of a muscle’s activation level, a plantar pressure-based system provides indirect feedback of a muscle’s activation level, focusing instead on the functional output of the muscle (i.e., plantar flexor force). With the EMG-based system, a user could raise the bar past their target level by contracting their soleus muscle without necessarily increasing plantar flexor force if the antagonist dorsiflexor muscle (i.e., tibialis anterior) was contracting at the same time. Our finding of increased co-contraction at the ankle with the EMG-based system relative to baseline indicates that this strategy was indeed adopted by our study participants. With the plantar pressure-based system, on the other hand, increasing co-contraction at the ankle would counter the desired output of increase plantar flexor force, likely explaining why we did not observe increases with this system relative to baseline. With the goal of using a biofeedback system to train improved neuromuscular control at the ankle for enhanced walking ability, the use of a plantar pressure-based system may result in a more functional outcome. This is corroborated by the observation reported in the literature that external focus of attention (i.e., increased force against an FSR) is superior to internal focus of attention (i.e., increased activation of the soleus) for functional motor learning [26]. To our knowledge, this is only the second study to evaluate the ability of an audiovisual biofeedback system to influence ankle plantar flexor muscle activity while walking in CP. The previous study focused on spatiotemporal and kinematic outcomes, and did not report the neuromuscular response (i.e., relative triceps surae activation) while walking with feedback [13]. The findings from the present study, therefore, are particularly enlightening, indicating that providing feedback in this manner to children and young adults with CP results in significantly increased neuromuscular recruitment while walking after only a few minutes of acclimation.

We observed statistically significant increases in mean and peak propulsive phase medial gastrocnemius activation with both biofeedback systems. The medial gastrocnemius serves a unique role relative to the soleus during walking, contributing more to forward propulsion [20]. It has been observed that individuals with CP have deficits in neuromuscular control of the medial gastrocnemii during the push-off phase of gait, leading to reduced ankle power and a slow and inefficient gait pattern [27]. Our finding of increased recruitment of this essential plantar flexor muscle, specifically during the propulsive phase of gait, supports the potential of this audiovisual biofeedback system to positively impact walking performance in this population.

There are instances where an EMG-based biofeedback system may be more advantageous than a plantar pressure-based system, such as barefoot walking or for a user whose orthotics are not compatible with a pressure-sensitive insole. This study, however, was motivated by the desire to test a more clinically accessible form of audiovisual biofeedback to improve neuromuscular control at the ankle while walking. From our anecdotal observations on the amount of time required to set up both systems, it became clear that a plantar pressure-based system was a less resource- and time-intensive process. For example, one can consider the complexity of placing the sensor for the two systems: for the plantar pressure-based system, it simply requires placing the sensor on the medial forefoot of the shoe insole. For the EMG-based system, one must utilize anatomical landmarks to locate an appropriate measurement spot of a muscle belly, which can be challenging for young children with low muscle volume; carefully prepare the skin interface by shaving hair and swabbing with alcohol; and carefully securing the sensor components to the skin to mitigate disturbances in the signal from movement artifacts. In addition, there are notable differences in the cost required for both systems, with materials required for a plantar pressure-based system coming in at a fraction of the price of the materials required for an EMG-based system. These practical differences, in conjunction with the findings from this study that a plantar pressure-based system is able to produce comparable or even more-favorable neuromuscular control at the ankle, support the future investigation of plantar pressure-based systems in clinical settings.

There are notable limitations of this study. First, we tested a relatively small sample of individuals with CP, which is an inherently heterogenous condition. Still, we observed relatively consistent increases in muscle activity across our participants with both audiovisual biofeedback systems. In addition, the primary aim of this study was to validate the plantar pressure-based system against an EMG-based system for a clinical population, and not necessarily to demonstrate any kind of training effect that may require greater statistical power. Second, this clinical validation was limited to individuals with CP. The primary outcome of improved plantar flexor recruitment, however, could be valuable to several patient populations with deficits in neuromuscular control at the ankle. Third, our validation was limited to treadmill walking, which was necessary to isolate the comparison between biofeedback systems and provide continuous visual feedback to the participants. Future work will explore the translation of the plantar pressure-based system to an overground walking context with audio-only biofeedback. Finally, this study did not include qualitative assessments of how the user experience compared between the two biofeedback systems, which should be explored in future applications of this plantar pressure-based system to confirm clinical viability.

## Conclusion

In conclusion, we demonstrated that a simple plantar pressure-based biofeedback system is capable of increasing functional recruitment of the ankle plantar flexor muscles in children and young adults with CP. We observed comparable or even more-favorable neuromuscular control at the ankle when using this system relative to direct EMG biofeedback. We also confirmed that these neuromuscular responses were not a result of compensatory walking patterns, with consistent lower limb kinematics compared to baseline walking. Future studies and clinical interventions should evaluate if functional training with this simple, low-cost system can result in lasting improvements in walking ability in CP and other patient populations.

## Supplementary Information

**Additional file 1.** Individual soleus activation curves for baseline, plantar pressure biofeedback, and EMG biofeedback walking conditions.

**Additional file 2.** Individual medial gastrocnemius activation curves for baseline, plantar pressure biofeedback, and EMG biofeedback walking conditions.

**Additional file 3.** Individual knee and ankle joint angles for baseline, plantar pressure biofeedback, and EMG biofeedback walking conditions.

## Data Availability

The datasets used and analyzed during the current study are available from the corresponding author on reasonable request.

## Abbreviations

CP: Cerebral palsy
EMG: Electromyography
FSR: Force sensitive resistor
GUI: Graphical user interface
GMFCS: Gross Motor Function Classification System
CCI: Co-contraction index
