# Supplementary material for: Under pressure: design and validation of a pressure-sensitive insole for ankle plantar flexion biofeedback during neuromuscular gait training
Source: J Neuroeng Rehabil. 2022 Dec 8;19:135. doi: 10.1186/s12984-022-01119-y (PMC9732996; doi:10.1186/s12984-022-01119-y)
Supplement: Supplementary file 2 — Additional file 2. Individual medial gastrocnemius activation curves for baseline, plantar pressure biofeedback, and EMG biofeedback walking conditions. [file 12984_2022_1119_MOESM2_ESM.docx]

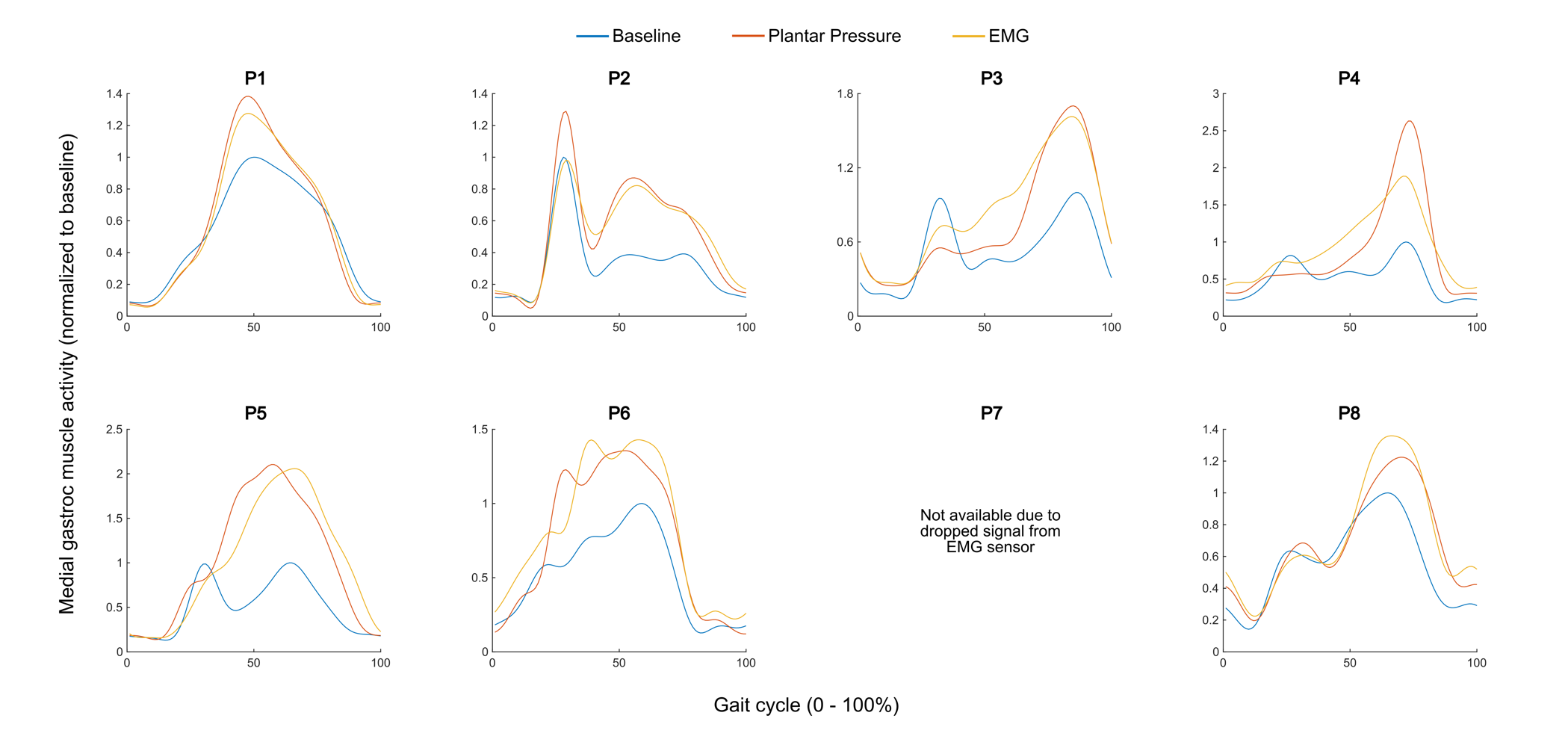


**Figure S2**. Mean medial gastrocnemius muscle activation curves, averaged over 20 gait cycles and normalized to peak baseline activation, across baseline (blue), plantar pressure-based biofeedback (orange), and EMG-based biofeedback (yellow) conditions.
