# Supplementary material for: Under pressure: design and validation of a pressure-sensitive insole for ankle plantar flexion biofeedback during neuromuscular gait training
Source: J Neuroeng Rehabil. 2022 Dec 8;19:135. doi: 10.1186/s12984-022-01119-y (PMC9732996; doi:10.1186/s12984-022-01119-y)
Supplement: Supplementary file 3 — Additional file 3. Individual knee and ankle joint angles for baseline, plantar pressure biofeedback, and EMG biofeedback walking conditions. [file 12984_2022_1119_MOESM3_ESM.docx]

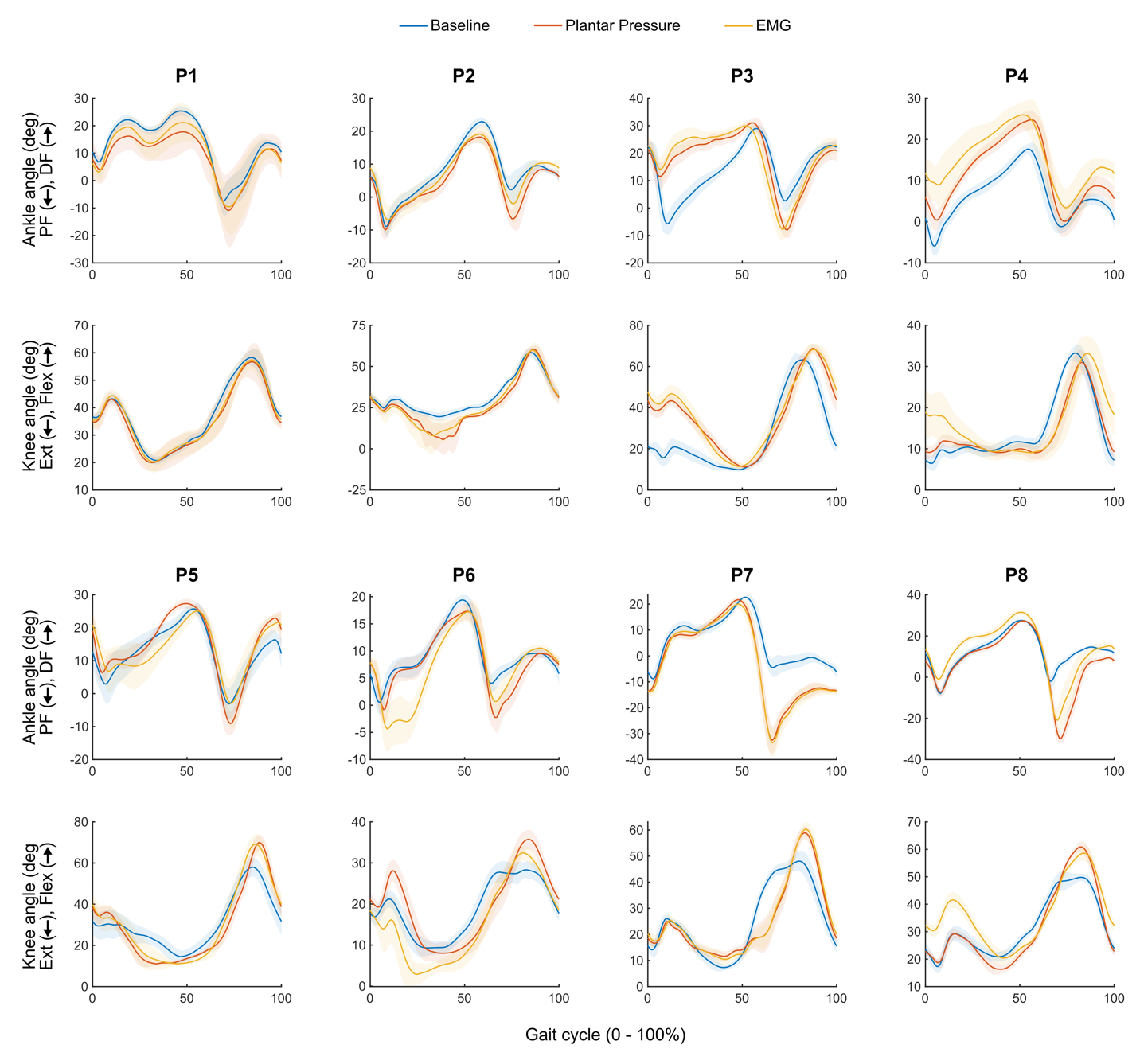


**Figure S3**. Mean ankle (rows 1 and 3) and knee (rows 2 and 4) joint angle curves, averaged over 20 gait cycles, across baseline (blue), EMG-based biofeedback (yellow), and plantar pressure-based biofeedback (orange) conditions. Shading indicates ± 1 standard deviation. Negative joint angles indicate extension.
